# High-performance SERS substrate based on hybrid structure of graphene oxide/AgNPs/Cu film@pyramid Si

**DOI:** 10.1038/srep38539

**Published:** 2016-12-07

**Authors:** Zhe Li, Shi Cai Xu, Chao Zhang, Xiao Yun Liu, Sai Sai Gao, Li Tao Hu, Jia Guo, Yong Ma, Shou Zhen Jiang, Hai Peng Si

**Affiliations:** 1 School of Physics and Electronics, Shandong Normal University, Jinan 250014, China; 2 Shandong Provincial Key Laboratory of Biophysics, College of Physics and Electronic Information, Dezhou University, Dezhou 253023, PR China; 3 Department of Orthopaedics, Qilu Hospital, Shandong University, 107 wenhuaxilu Street, Jinan 250012, China

## Abstract

We present a novel surface-enhanced Raman scattering (SERS) substrate based on graphene oxide/silver nanoparticles/copper film covered silicon pyramid arrays (GO/AgNPs/PCu@Si) by a low-cost and simple method. The GO/AgNPs/PCu@Si substrate presents high sensitivity, good homogeneity and well stability with R6G molecules as a probe. The detected concentration of Rhodamine 6 G (R6G) is as low as 10^−15^ M. These sensitive SERS behaviors are also confirmed in theory via a commercial COMSOL software, the electric field enhancement is not only formed between the AgNPs, but also formed between the AgNPs and Cu film. And the GO/AgNPs/PCu@Si substrates also present good property on practical application for the detection of methylene blue (MB) and crystal violet (CV). This work may offer a novel and practical method to facilitate the SERS applications in areas of medicine, food safety and biotechnology.

Surface-enhanced Raman scattering (SERS), has been widely used as a fingerprint method for ultra-sensitive and selective detection in biology and chemistry. The SERS signals can be amplified to 10^12^–10^15^ times when the target molecules reside on the proper position between metal nanostructures (generally called “hot spots”). The structure and material of the substrate play crucial part in the SERS performance. At present, it is widely accepted that the electromagnetic mechanism (EM) and chemical mechanism (CM) are the mechanisms of SERS^1^. The EM originates from the dramatic increase in the local electromagnetic field, which can boost the pristine Raman signal by 10^8^ times or more^2^,^3^. It has been demonstrated that the EM enhancement of SERS can be introduced by metal nanoparticles (Au, Ag and Cu) or metal nanoparticle and metal film^4^,^5^,^6^. The CM caused by charge transfer between the target molecules and the substrate, usually has a minor enhancement factor of 10-10^2^^7^. Although the EM plays the dominate role, the CM can reduce the background florescence effectively. Recently, the researches have found that the graphene can introduce the enhancement based on the CM. Graphene, a single layer of sp^2^ carbon network arranged in a perfect honeycomb lattice, has been extensively investigated because of its excellent mechanical and electrical properties^8^,^9^. Compared with the graphene, graphene oxide (GO) has superior bio-compatibility and chemical stability due to the active oxygen sites^10^,^11^, which can notably enhance the graphene-metal/molecule binding, depress the fluorescence background, and increase the Raman/fluorescence signal-to-noise ratio.

Besides the active materials, the microstructures of the SERS substrates also play a critical role on the SERS signal. It has been proved that the porous Si (PS) can increase the amount of the effective hot spots and further enhance the sensitivity of the SERS signals with the assist of the large specific area and nanoporous governable structure^12^,^13^,^14^,^15^,^16^. Recently, some groups have reported different SERS substrates based the PS, such as PS decorated with Au nanoparticles^12^,^17^ and Ag-coated Si nanoporous^18^,^19^. Very recently we found that the local electric field enhancement on the CuNPs/Cu foil is stronger than that on CuNPs/SiO_2_ substrate. It has been proven that local surface plasmon can be formed between the noble metal nanoparticles and metal substrate^20^. Considering the pyramid Si (PSi) is nonmetal, we attempt to deposit Cu film on the PSi and form a metal pyramid with a simple and low-cost method.

In this paper, based on the above advantages, we combine the GO, Ag nanoparticles (AgNPs), Cu film and PSi forming the GO/AgNPs/PCu@Si substrate. The GO/AgNPs/PCu@Si substrate shows the following advantages: (1) The PSi offers more hot spots. (2) We make use of the morphology characteristic of PSi to fabricate a layer of pyramid Cu film, the local electric field enhancement can formed between the pyramid Cu film and the AgNPs thereby to obtain more sensitive SERS substrate. (3) Besides the above advantages of the GO, it can also prevent the oxidation of the metal nanoparticles, more molecules can effectively absorb on the hot spots and thus more stable and sensitive Raman signals are expected. What’s more, the GO makes molecules distribute more uniformly, it leads to higher linearity. Using the proposed GO/AgNPs/PCu@Si substrate, the sensitive, homogeneous and stable SERS signals of R6G and methylene blue (MB) was successfully collected. This work indicates that the GO/AgNPs/PCu@Si substrate have great potential for the practical application in biological sensing and other biotechnology.

## Experimental

**Figure 1:**
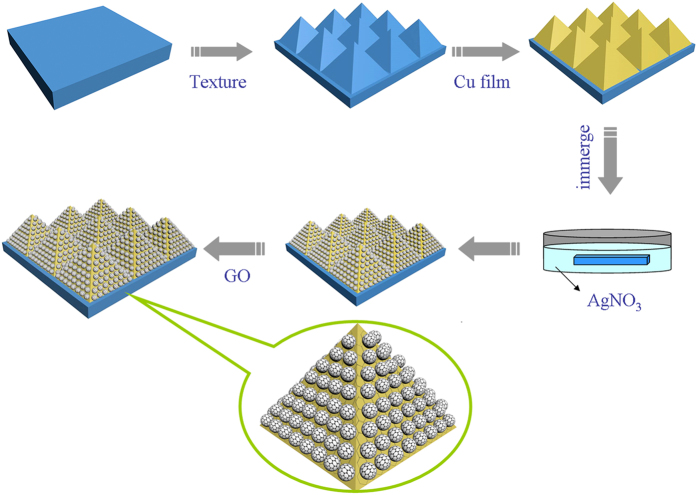
Schematic representation of the preparation procedure of the GO/AgNPs/PCu@Si substrate.

Figure 1 schematically illustrates the simple process for the synthesis of GO/AgNPs/PCu@Si substrate PSi substrate (boron-doped single crystal silicon) was fabricated by using wet texturing technology with the assist of NaOH^21^. The Cu film was synthesized on the PSi by thermal evaporation. Then these prepared Cu film coverd PSi (PCu@Si) were respectively immersed into 1 mM AgNO_3_ solution for 3, 4, 5, 6 and 7 min to fabricate the AgNPs/PCu@Si substrate. After the AgNPs/PCu@Si was rinsed by deionized water, and dried in the N_2_ atmosphere. In order to control the number of layers of the GO, we choose to control the concentration and volume of the GO suspension, then the 50 μL and 0.5 mg/mL GO (obtained using the modified Hummers method^22^) suspension was deposited on the surface of the AgNPs/PCu@Si substrate by a dip-coating method. The prepared GO/AgNPs/PCu@Si substrate sealed in the nitrogen atmosphere was ready for the SERS measurements. All the pyramid-Si substrates in the experiment were cleaned by acetone, alcohol and deionized water until employed.

The surface morphology of the GO/AgNPs/PCu@Si substrate was characterized by scanning electron microscope (SEM, Zeiss Gemini Ultra-55) and atomic force microscope (AFM, Park XE-100) in the noncontact mode. The transmission electron microscopy (TEM) is carried out by a transmission electron microscopy system (Hitachi H-800). SERS experiments were carried out with a Horiba HR Evolution 800 Raman spectrometer with laser wavelength at 473, 532 and 633 nm. All the spectra were collected under the same conditions (integration time: 4 s), the excitation laser spot was about 1 μm, and the effective power of the laser source was kept at 50 mW.

## Results and Discussion

**Figure 2:**
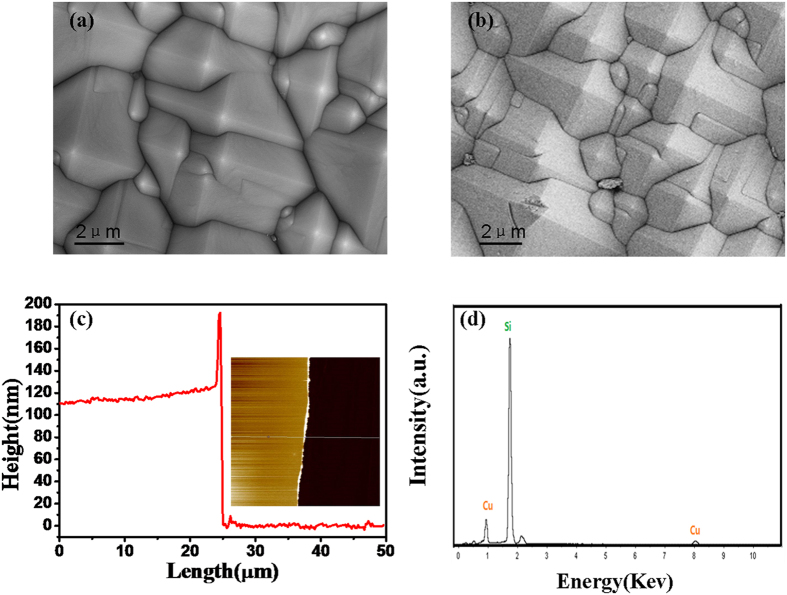
(**a**) SEM image of PSi. (**b**) SEM image of PCu@Si. (**c**) AFM image of the Cu film on the flat Si substrate. (**d**) The EDS spectrum of PCu@Si.

Figure 2a shows the surface morphology of the PSi substrate, where one can observe that the regular pyramid arrays are relatively uniform. As shown in Fig. 2b, the PSi is covered by Cu film completely after the treatment of thermal evaporation and the Cu film also presents the pyramid shape. Because of the rough surface of the PSi, it is difficult to measure the thickness of the Cu film on the PSi substrate. Therefore, we deposited the Cu film on flat Si substrate by thermal evaporation under the same conditions to detect the thickness of the Cu film. The Fig. 2c shows AFM image of the Cu film on flat Si substrate. As we can see from the line profile of the Cu film, the thickness of the Cu film is 110 nm. What’s more, based on the EDS spectrum of the PCu@Si as shown in Fig. 2d, we can draw a conclusion that the Cu film has been fabricated on pyramid-Si successfully and continuously.

**Figure 3:**
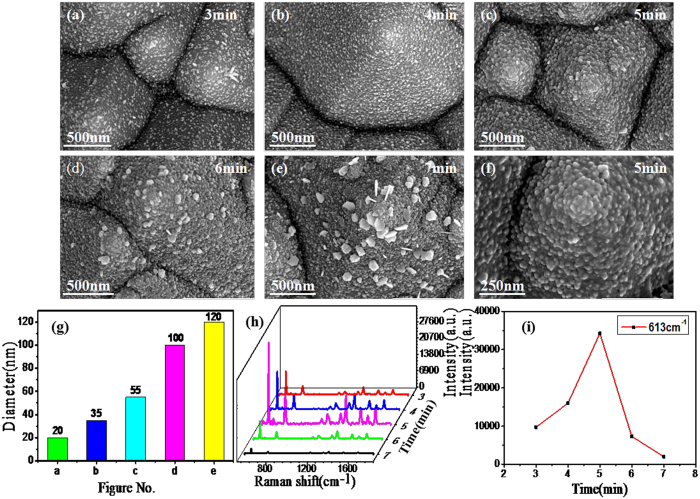
(**a–e**) SEM image of the different reaction time (3-7 min) of the AgNPs/PCu@Si substrates. (**f**) is the magnified image of the sample in (**c**). (**g**) is histogram of the diameter of Ag nanoparticles from Fig. 3(**a**) to (**e**). (**h**) SERS spectra of R6G (10^−8^ M) on those AgNPs/PCu@Si substrates fabricated with different reaction time (3-7 min). (**i**) The intensity of the R6G at 613 cm^−1^ peak changes as a function of replacement reaction time.

The Fig. 3a–e present the SEM images of the AgNPs/PCu@Si substrates with different reaction time (3-7 min). As we can see, the size of AgNPs becomes larger with the reaction time increase, and meanwhile, the gaps between the nanoparticles get smaller. When the reaction time further increases to 6 min, the AgNPs begin clustering. This phenomenon becomes acute with the reaction time of 7 min. Figure 3g is histogram of the diameter of AgNPs from Fig. 3a to e. And as shown in Fig. 3g, the diameters of the AgNPs are 20, 35, 55, 100 and 120 nm, respectively. In order to examine the SERS activity of the AgNPs/PCu@Si substrate fabricated with different reaction time, we chose the R6G as probe molecules. Corresponding SERS spectra of R6G (10^−8^ M) on those AgNPs/PCu@Si substrates fabricated with different reaction time were obtained as shown in Fig. 3h. The characteristic Raman peaks of R6G at 613, 774, 1185, 1311, 1362, 1506 and 1647 cm^−1^ are observed in Fig. 3h. The peaks located at 613 cm^−1^ is corresponded to the C-C-C in-plane vibration of the R6G molecular^23^. The peaks located at 774 and 1185 cm^−1^ can be respectively attributed to the out-of-plane vibration and in-plane vibration of C-H bonds. The peaks observed at 1311, 1362, 1506 and 1647 cm^−1^ can be assigned to the aromatic C-C stretching vibration mode. As shown in Fig. 3i, the intensity changes as a function of replacement reaction time. Obviously, the intensity at 613 cm^−1^ peak increases with the replacement reaction time increase from 3 to 5 min and saturates at the 5 min, then the intensity decreases with the replacement reaction time increase from 5 to 7 min. Through this process, we draw a conclusion that the AgNPs/PCu@Si substrate possesses optimum SERS activity with a replacement reaction time of 5 min. Therefore, we choose the substrate with 5 mins reaction time as the object to further research. First of all, we need to detect the thickness of the Cu film for target substrate. On account of the same reason, we detect the Cu film reacting with AgNO_3_ solution for 5 min on flat Si substrate via AFM. As shown in Supplementary Fig. S1, the AFM image reveals the thickness of Cu film is about 70 nm, and the fold line indicates that there are indeed nanoparticles on the Cu film. Supplementary Fig. S1 shows the EDS spectrum of the AgNPs/PCu@Si with a replacement reaction time of 5 min, where the existence of Ag element demonstrates that the Ag element has been fabricated expectedly.

**Figure 4:**
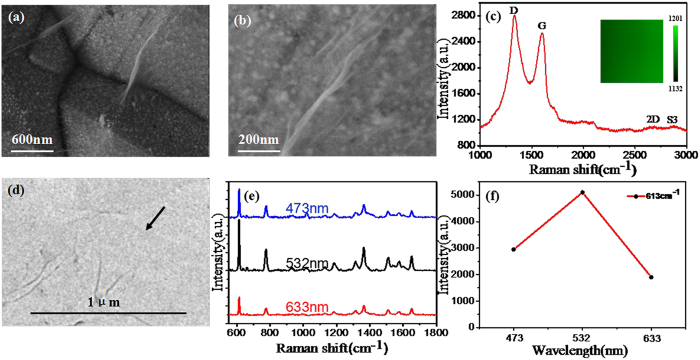
(**a**) SEM image of GO/AgNPs/PCu@Si substrate. (**b**) is the SEM image of GO/AgNPs/PCu@Si substrate under a larger magnification. (**c**) Raman spectrum of the GO and the Raman mapping in the inset. (**d**) TEM of the GO film. (**e**) Raman spectrum of R6G with the concentration of 10^−12^ M on the GO/AgNPs/PCu@Si substrate under the laser wavelength with 473, 532 and 633 nm respectively. (**f**) The intensity of the R6G at 613 cm^−1^ peak changes as a function of the laser wavelength.

Based on the above experiment results, we combine the optimum AgNPs/PCu@Si SERS substrate with the GO with dip-coating method. Figure 4a and b are the SEM image of the GO/AgNPs/PCu@Si substrate under different magnification. Obviously, some wrinkles are observed on the surface of the GO/AgNPs/PCu@Si substrate, and the AgNPs/PCu@Si substrate is almost covered by the GO thin layer. In order to further demonstrate the existence of the GO film on the GO/AgNPs/PCu@Si substrate, Raman spectra were obtained. As shown in Fig. 4c, the D, G, 2D and S3 bands of GO are clearly observed. The D band (1360 cm^−1^) is assigned to the ring vibration symmetrical breathing mode and associated with the defects caused by the attachment of hydroxyl and epoxide groups. The G band (1595 cm^−1^) is assigned to the first-order scattering of the in-plane optical phonon E_2g_ mode and the 2D band (2722 cm^−1^) is assigned to the second-order process involving two phonons with opposite momentum. The S3 band (2930 cm^−1^) is caused by the imperfect activated grouping of phonons. The Raman spectra clearly demonstrate that high-quality GO films have coated on the AgNPs/PCu@Si. To evaluate the uniformity of GO, Raman mapping of D-peak to G-peak of GO on AgNPs/PCu@Si substrates were implemented over 20 μm × 20 μm area as shown in the inset of Fig. 3c. From the color scale in the inset, the intensity of D-peak to G-peak shows a small fluctuation from 1132 to 1201, the result indicates that the GO have a relatively uniform structure in a large area. The TEM image of the GO is shown in Fig. 4d, an edge can be seen clearly at the black arrow, the color turn darker with the increase of the layers of GO film. The TEM results indicate that the GO possesses a relatively uniform thickness. And as shown in Fig. 4e, we detected the R6G with the concentration of 10^−12^ M on the GO/AgNPs/PCu@Si substrates under the laser wavelength with 473, 532 and 633 nm respectively, we can ensure that the intensity of the Raman spectrum is the highest under the laser wavelength with 532 nm. And in order to compare the intensity of the Raman spectrum under the three different laser wavelengths more visually, we take the intensity of the R6G at 613 cm^−1^ peak changes as a function of the laser wavelength, as shown in Fig. 4f. And the main reason of the phenomenon we concluded is that the absorption band of R6G on the AgNPs is nearby the 532 nm, and the R6G molecular resonances play the main role^23^.

**Figure 5:**
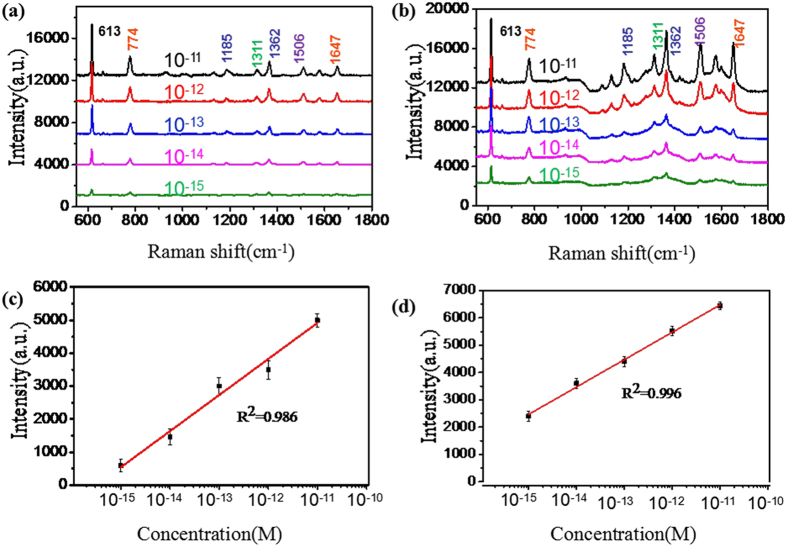
(**a**) and (**b**) are respectively Raman spectra of R6G on the AgNPs/PCu@Si and the GO/AgNPs/PCu@Si substrate with concentration from 10^−10^ to 10^−15^ M. (**c**) and (**d**) are respectively the Raman intensity of R6G peaks at 613 cm^−1^ as a function of the molecular concentration on the AgNPs/PCu@Si and GO/AgNPs/PCu@Si, in log scale.

**Figure 6:**
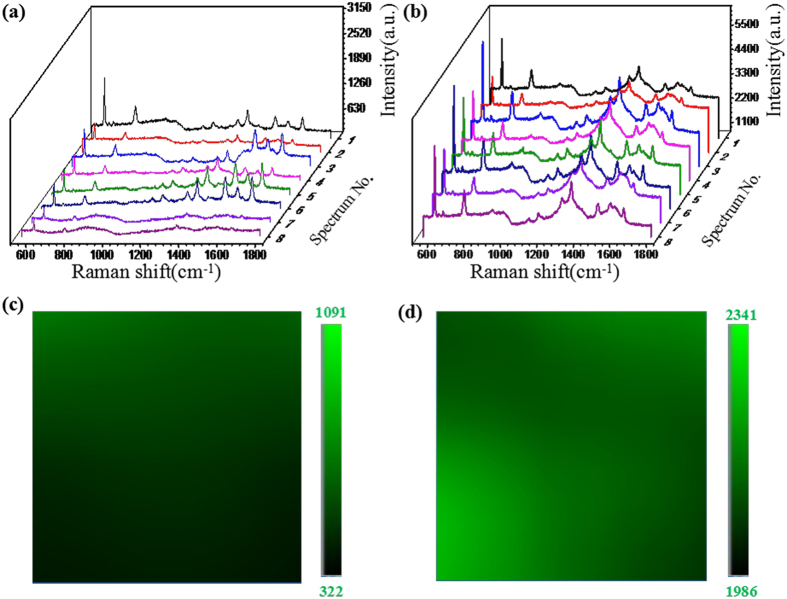
(**a**) and (**b**) are respectively SERS spectra of the R6G with concentration of 10^−15^ M from 8 spots on the AgNPs/PCu@Si and GO/AgNPs/PCu@Si substrate. (**c**) and (**d**) are the Raman mapping at 613 cm^−1^ of R6G molecules at 10^−15^ M dispensed on the GO/AgNPs/PCu@Si substrates and the AgNPs/PCu@Si substrates respectively.

To further investigate the SERS activity of our proposed GO/AgNPs/PCu@Si substrate, we compared the SERS performance of the GO/AgNPs/PCu@Si substrate with that of the AgNPs/PCu@Si substrate. The R6G aqueous solutions with varied concentrations were used as the probe molecule. Figure 5a and b show the Raman spectra of R6G on the AgNPs/PCu@Si and GO/AgNPs/PCu@Si substrate respectively with various concentrations from 10^−10^ to 10^−15^ M. After the coating of GO film, it is drastically distinct that the intensities of SERS spectra from the GO/AgNPs/PCu@Si are much stronger than those of AgNPs/PCu@Si. The Raman spectra of R6G with concentration of 10^−15^ M can be easily detected on the GO/AgNPs/PCu@Si substrate, where the intensity of the peaks at 613 cm^−1^ is about 2 times stronger than that of the AgNPs/PCu@Si substrate. This phenomenon can be attributed to the excellent bio-compatibility of GO, which can serve as a superior molecule-enricher and enhance the Raman signal^24^,^25^,^26^,^27^,^28^. Because the higher intensity of the Raman spectra of R6G compared with that of the GO, the Raman spectra of the GO become few obvious, but it still can be seen in the Fig. 5b at 1360 and 1595 cm^−1^ compared with Fig. 5a, there are two bulges obviously. Figure 5c and d show respectively the Raman intensity of R6G peaks at 613 cm^−1^ as a function of the molecular concentration on the AgNPs/PCu@Si and GO/AgNPs/PCu@Si, in log scale, where the value of *R*^2^ reaches 0.986 and 0.996 indicating the linearity of the latter is superior to the former. In the latter case, the GO acts as the excellent adsorbent towards organic molecules, and leads to the R6G molecules uniformly distribute. As the Fig. 6a and 6b shown, we randomly detected the SERS spectra of the R6G with the same concentration from 8 spots on the same AgNPs/PCu@Si and GO/AgNPs/PCu@Si substrate. It’s obvious that the homogeneity of SERS signal on the GO/AgNPs/PCu@Si is better than that on the AgNPs/PCu@Si substrate. To evaluate the uniformity of GO/AgNPs/PCu@Si substrates, the Raman mapping of 613 cm^−1^ peak of R6G with the concentration of 10^−15^ M was implemented over 20 μm × 20 μm area on GO/AgNPs/PCu@Si substrates and AgNPs/PCu@Si substrates respectively, as shown in the Fig. 6c and d. From the color scale in the figures, the GO/AgNPs/PCu@Si substrates show a smaller fluctuation from 1986 to 2341 compared with that on the AgNPs/PCu@Si substrates from 322 to 1091, therefore we concluded that the GO/AgNPs/PCu@Si substrates have possesses well uniformity for SERS signal in a large area. The relatively well uniformity of the GO/AgNPs/PCu@Si substrates can be ascribed to the well distributed AgNPs and the existence of GO films. The well distributed AgNPs on the Cu film surface can achieve the well distributed hot spots and the GO films covering both AgNPs and spaces can make the probe molecule effectively absorbed around the hot spots^29^. For the former case, as the absence of the GO film, the molecules will distribute unevenly on the AgNPs/PCu@Si substrate, which will lead to the weak homogeneity of SERS signal. The enhancement factor (EF) for GO/AgNPs/PCu@Si substrates was calculated according to the following equation


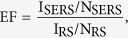


where I_SERS_ and I_RS_, respectively, represent the peak intensities of the SERS spectra and the normal Raman spectra, N_SERS_ and N_RS_ are, respectively, the numbers of molecules on the substrates within the laser spot. According to the above equations, in the experiments, the EF of R6G with a concentration of 10^−15^M is calculated to be 6.7 × 10^11^ for the AgNPs/PCu@Si substrate, 2 × 10^12^ for the GO/AgNPs/PCu@Si substrate. Compared with the AgNPs/PCu@Si substrate, the GO/AgNPs/PCu@Si substrates exhibit an enhancement of about 3 times in the EF values resulting from the CM of the GO. The EF of the GO/AgNPs/PCu@Si substrate is 1.7 × 10^3^ times larger than that of the Au@Ag/3D-Si substrate^30^, 6.06 × 10^3^ times larger than the G/AgNP array substrates^31^. The excellent SERS sensitivity of the GO/AgNPs/PCu@Si substrate can be attributed to the combined action of the pyramid of the PCu@Si, the coupling of GO and plasmonic AgNPs, and the local electric field enhancement between the AgNPs and Cu film.

**Figure 7:**
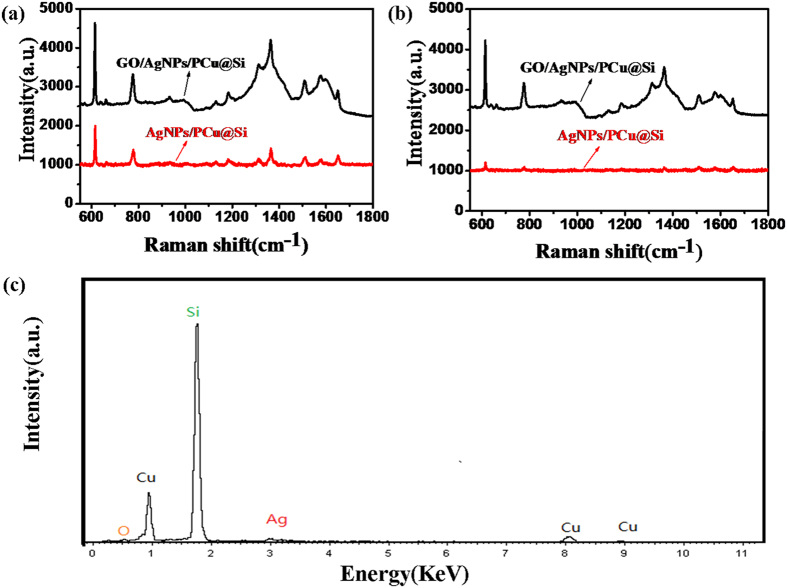
(**a**) and (**b**) are respectively the SERS spectra of the R6G on the GO/AgNPs/PCu@Si and AgNPs/PCu@Si SERS substrate without and with the oxidation treatment. (**c**) The EDS result of the AgNPs/PCu@Si substrate after the expose of oxygen.

Furthermore we measure the stability of the GO/AgNPs/PCu@Si and AgNPs/PCu@Si SERS substrate by subjecting it to aerobic exposure for 15 days. As shown in Fig. 7a and 7b, what should be noticed is that, after the oxidation treatment, the intensity of R6G with a concentration of 10^−15^ M on the GO/AgNPs/PCu@Si substrate is almost invariant, indicating that the GO/AgNPs/PCu@Si substrate possesses excellent antioxidant stability. On the contrary, the intensity of the R6G with a concentration of 10^−15^ M on the AgNPs/PCu@Si substrate decreases obviously after being treated with oxidation. The decrease of the intensity may be due to the oxidation of AgNPs as it is greatly impressionable to oxidation, which can be confirmed by the EDS result in Fig. 7c.

**Figure 8:**
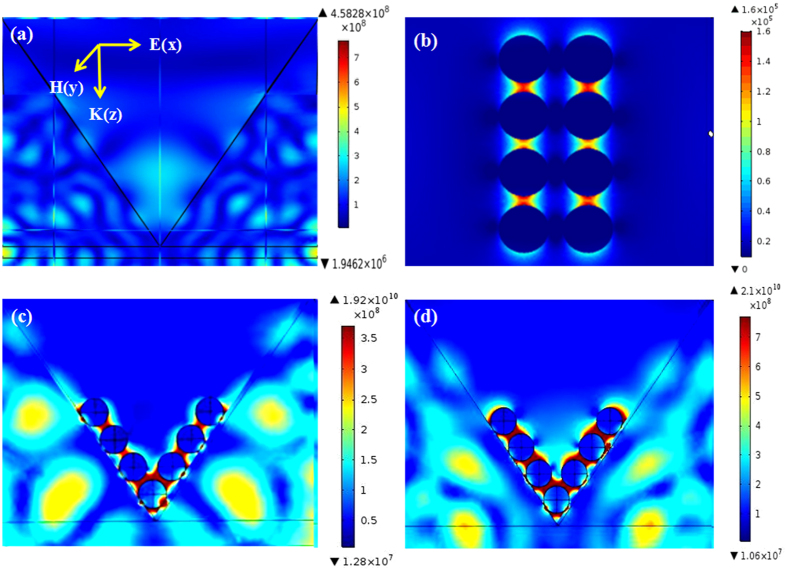
(**a**) The x-z views of the electric field distribution on PSi substrate. (**b**) The x-y views of theElectric field distribution on 55 nm AgNPs/Si structure with gaps of 10 nm. And the definitions of the geometrical parameters in the inset. E–electric field; H–magnetic field; k–direction of light propagation. (**c**) The x-z views of the electric field distribution on 55 nm AgNPs/PSi structure with gaps of 10 nm. (**d**) The x-z views of the electric field distribution on 55 nm AgNPs/PCu@Si structure with gaps of 10 nm.

In order to further identify the effect of the PCu film for the enhanced electric field and better understand the SERS enhancement mechanism of the GO/AgNPs/PCu@Si sbustrate. We calculated and analyzed the local electric field properties of the PSi, AgNPs/Si, AgNPs/PSi, AgNPs/PCu@Si structure using commercial COMSOL software. The Fig. 8a shows the x-z views of the electric field distribution on the PSi sample with incident light wavelength 532 nm and as is shown in the inset, the definitions of the geometrical parameters are provided: E(x), H(y) and K(z) are the electric field (the polarization direction of laser), magnetic field and direction of light propagation respectively. It’s obvious that electric field is weak, just as we have discussed above, the EM is from metal nanoparticles, once attaching the AgNPs on the surface, it can effectively amplify and increased electrical field intensity of the plasmonic resonance. In order to prove that the superiority of the PSi compared to flat Si, we model AgNPs/Si and AgNPs/PSi structure, respectively. As shown in Fig. 8b and c, we set the diameter of AgNPs as 55 nm and gaps as 10 nm. It can be seen clearly that the magnitude of electrical field of AgNPs/PSi is much larger than that of AgNPs/Si substrate. And then, we build the theoretical model of the AgNPs/PCu@Si, we set the diameter of AgNPs as 55 nm and gaps as 10 nm and the thickness of the Cu film as 70 nm. As shown in Fig. 8d, the electrical field is stronger than that of AgNPs/PSi, the reason for this phenomenon is that local surface plasmon will be formed between AgNPs and Cu film. For the AgNPs/Si case, while, the local surface plasmon can not be formed between nanoparticles and PSi substrate. Therefore, the excellent SERS behaviors of the Ag/PCu@Si substrate can contributed to the following points: (1) the pyramidal structure of the PSi substrate can be used as the amplifier for incident light and introduce large electrical field intensity of the plasmonic resonance. (2) The PCu film can provide an extra electrical field enhancement due to the interaction with AgNPs. Based on these theoretical results, we can conclude that the GO/AgNPs/PCu@Si SERS substrate with higher sensitivity will be realized by further optimizing its structure.

**Figure 9:**
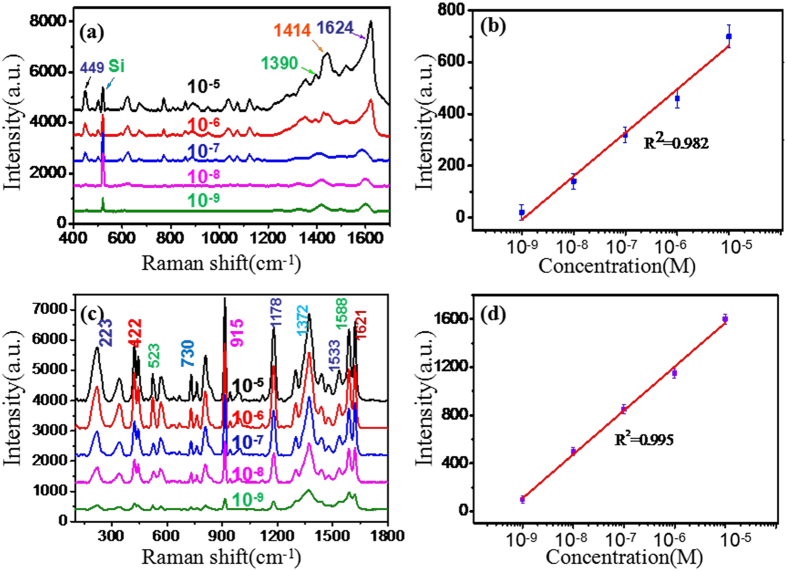
(**a**) Raman spectra of MB with concentration from 10^−5^ to 10^−9^ M on the GO/AgNPs/PCu@Si substrate with 5 min replacement reaction. (**b**) The Raman intensity of MB at 449 cm^−1^ as a function of the molecular concentration on the GO/AgNPs/PCu@Si substrate, in log scale. (**c**) Raman spectra of CV with concentration from 10^−5^ to 10^−9^ M on the GO/AgNPs/PCu@Si substrate with 5 min replacement reaction. (**d**) The Raman intensity of CV at 223 cm^−1^ as a function of the molecular concentration on the GO/AgNPs/PCu@Si substrate, in log scale.

To investigate the feasibility of the GO/AgNPs/PCu@Si substrates in practical application, the MB in deionized water with concentration of from 10^−5^ to 10^−9^ M were tested on GO/AgNPs/PCu@Si substrates. MB dye can cause eye burns, which may be responsible for permanent injury to the eyes of human and animals. Once inhalation, it can give rise to short periods of rapid and difficult breathing, if ingestion through the mouth it will produce a burning sensation and may cause nausea, vomiting, profuse sweating, mental confusion, painful micturition, and methemoglobinemia^32^. Therefore, achieving the effective detection of the MB is very significant for the health of human. In Fig. 9a, the characteristic Raman peaks of MB at 449, 670, 860, 1030, 1150, 1390, 1442 and 1617 cm^−1^ are observed. Among these peaks, the peak at 449 cm^−l^ can be attributed to the C-N-C skeletal deformation mode G(CNC), the peak at 1390 and 1442 cm^−l^ can respectively, be attributed to the symmetric and asymmetric CN stretches (v_sym_(CN) and v_asym_(CN)), and the peak at 1624 cm^−l^ can be attributed to the ring stretch (v(CC)). To demonstrate the capability of the quantitative detection of MB, the linear fit calibration curve (*R*^*2*^ = 0.982) is illustrated in Fig. 9b. A good linear response of SERS is obtained from 10^−3^ to 10^−9^ M. What’s more, we also detected the CV on GO/AgNPs/PCu@Si substrates with concentration of from 10^−5^ to 10^−9^ M. The CV is also a kind of dyes, which is used to control fungi and intestinal parasites in humans, as an antimicrobial agent on burn victims, to treat umbilical cords of infants, for the treatment of long-term vaginal candidosis, for various purposes in veterinary medicine, etc.^33^,^34^. The Fig. 9c shows the characteristic Raman peaks of CV at 223, 422, 523, 730, 915, 1178, 1372, 1533, 1588 and 1621 cm^−1^, among these peaks, the peaks at 223, 422, 523 (915), 730, 1178 and 1372 cm^−1^ can be assigned to the breathing of central bonds, Ph-C^+^-Ph bend, ring skeletal vib of radical orientation, ring C-H bend, ring skeletal vib of radical orientation, N-phenyl stretching, respectively. And the peaks at 1533, 1588 and 1621 cm^−1^ can be assigned to the ring C-C stretching^35^. And the linearity is also provided in Fig. 9d, the linear fit calibration curve (R^2^ = 0.995) shows the good linear response. By detecting the MB and CV successfully, the result indicates that a great potential application of the GO/AgNPs/PCu@Si substrates to detect other analytes in real biological systems can be well achieved.

## Conclusions

We have fabricated a SERS-active substrate based on GO/AgNPs/PCu@Si using a simple and low-cost method. Based on the SERS results, we can obtain SERS signals with high sensitivity, homogeneity and stability using R6G molecules as a probe. The SERS behaviors and the crucial role of PCu film for the electric field enhancement are also confirmed in theory via a commercial COMSOL software. What’s more, the GO/AgNPs/PCu@Si substrates also exhibit a great prospect of practical application for the detection of MB and CV. This work may offer a novel and practical method to facilitate biosensing applications.

## Additional Information

**How to cite this article**: Li, Z. *et al*. High-performance SERS substrate based on hybrid structure of graphene oxide/AgNPs/Cu film@pyramid Si. *Sci. Rep.* **6**, 38539; doi: 10.1038/srep38539 (2016).

**Publisher’s note:** Springer Nature remains neutral with regard to jurisdictional claims in published maps and institutional affiliations.

## Supplementary Material

Supplementary Information
