# Supplementary material for: High-performance SERS substrate based on hybrid structure of graphene oxide/AgNPs/Cu film@pyramid Si
Source: Sci Rep. 2016 Dec 7;6:38539. doi: 10.1038/srep38539 (PMC5141445; doi:10.1038/srep38539)
Supplement: Supplementary Information [file srep38539-s1.doc]

**High-performance SERS substrate based on hybrid structure of graphene oxide/AgNPs/Cu film@pyramid Si**

**Zhe Li^1^, Shi Cai Xu^2^, Chao Zhang^1^, Xiao Yun Liu,^1^ Sai Sai Gao^1^, Li Tao Hu^1^，Jia Guo^1^， Yong Ma^1^, Shou Zhen Jiang^1*^, Hai Peng Si^3^**

*1 School of Physics and Electronics, Shandong Normal University, Jinan 250014,* *China*

*2 Shandong Provincial Key Laboratory of Biophysics,College of Physics and Electronic Information, Dezhou University, Dezhou 253023, PR China*

*3 Department of Orthopaedics, Qilu Hospital, Shandong University, 107 wenhuaxilu Street, Jinan 250012, China*

*^*^ Corresponding author*: [*jiang_sz@126.com*](mailto:jiang_sz@126.com)


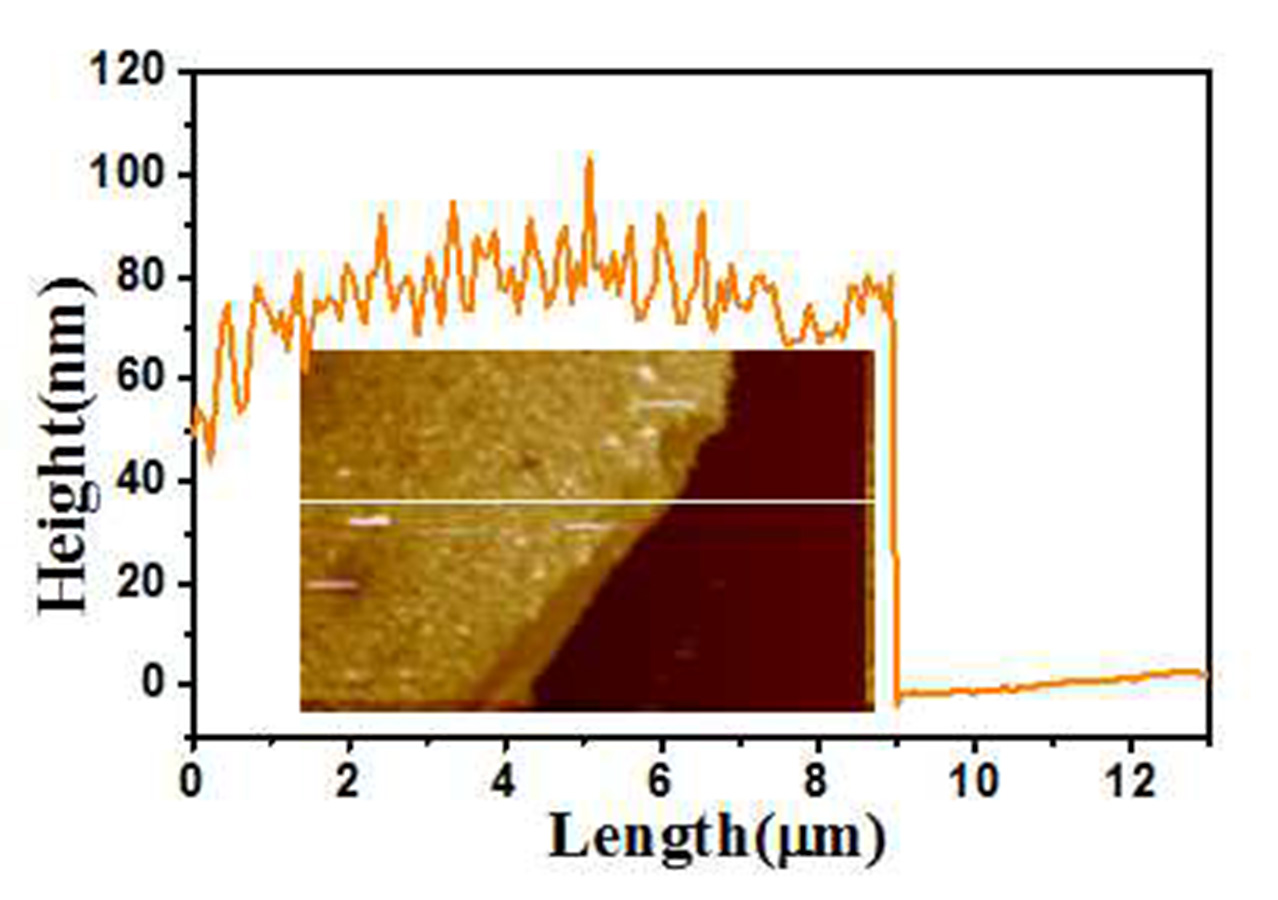


Supplementary Figure S1. The AFM image of Cu film react with AgNO_3_ solution for 5 min on flat Si substrate.


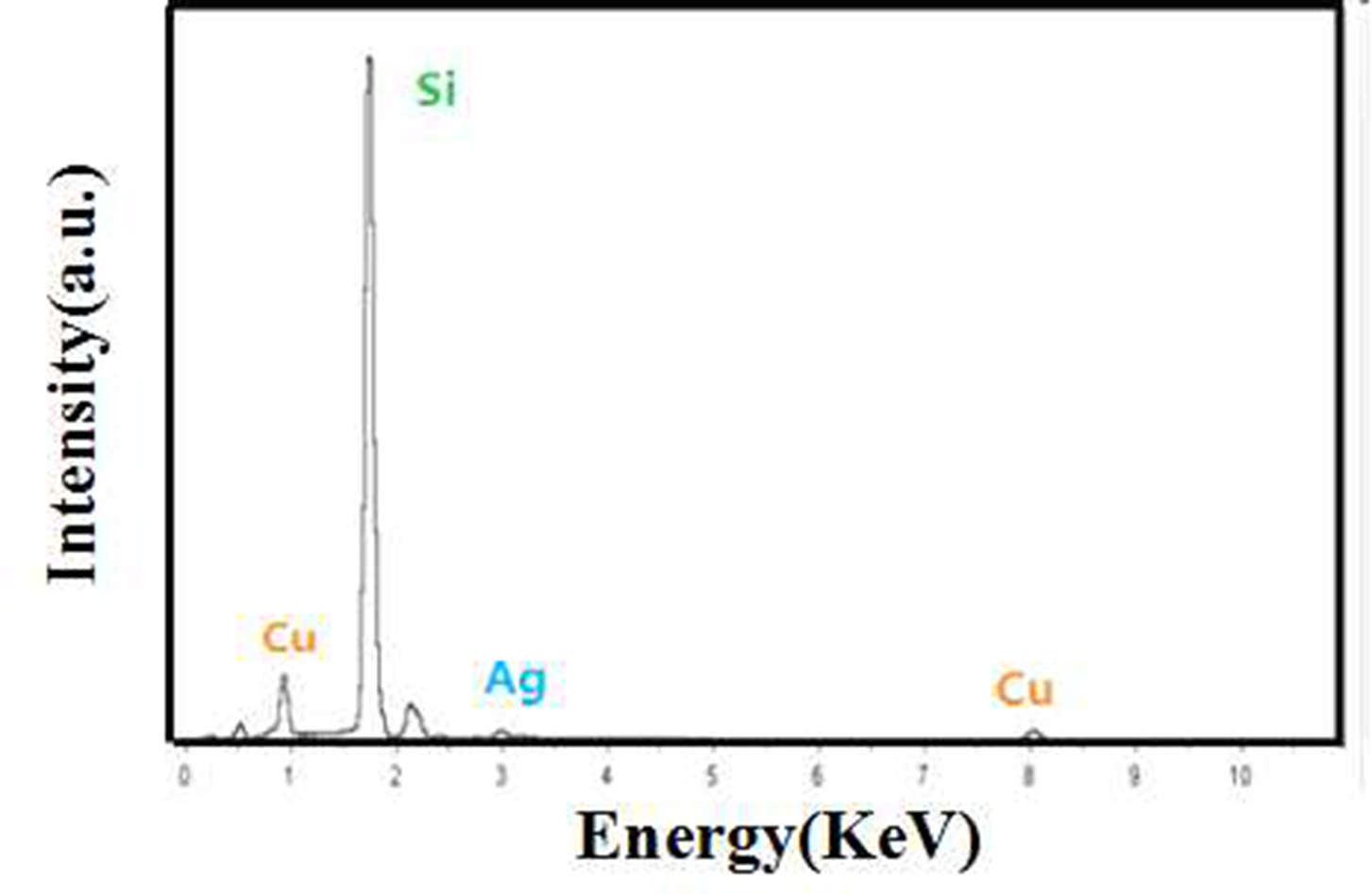


Supplementary Figure S2. The EDS spectrum of AgNPs/PCu@Si.
